# A novel Fibroblast Growth Factor Receptor family member promotes neuronal outgrowth and synaptic plasticity in *Aplysia*

**DOI:** 10.1007/s00726-014-1803-2

**Published:** 2014-07-25

**Authors:** Daniela D. Pollak, Bui Quang Minh, Ana Cicvaric, Francisco J. Monje

**Affiliations:** 1 Department of Neurophysiology and Neuropharmacology, Medical University of Vienna, Center for Physiology and Pharmacology, Schwarzspanierstrasse 17, 1090 Vienna, Austria; 2 Center for Integrative Bioinformatics Vienna, Max F Perutz Laboratories, University of Vienna, Medical University of Vienna, Vienna, Austria

**Keywords:** Fibroblast Growth Factor Receptor, Leucine-Rich Repeat, Nervous system, Neuronal outgrowth, Evolutionary divergence, Synaptic plasticity

## Abstract

Fibroblast Growth Factor (FGF) Receptors (FGFRs) regulate essential biological processes, including embryogenesis, angiogenesis, cellular growth and memory-related long-term synaptic plasticity. Whereas canonical FGFRs depend exclusively on extracellular Immunoglobulin (Ig)-like domains for ligand binding, other receptor types, including members of the tropomyosin-receptor-kinase (Trk) family, use either Ig-like or Leucine-Rich Repeat (LRR) motifs, or both. Little is known, however, about the evolutionary events leading to the differential incorporation of LRR domains into Ig-containing tyrosine kinase receptors. Moreover, although FGFRs have been identified in many vertebrate species, few reports describe their existence in invertebrates. Information about the biological relevance of invertebrate FGFRs and evolutionary divergences between them and their vertebrate counterparts is therefore limited. Here, we characterized ApLRRTK, a neuronal cell-surface protein recently identified in *Aplysia*. We unveiled ApLRRTK as the first member of the FGFRs family deprived of Ig-like domains that instead contains extracellular LRR domains. We describe that ApLRRTK exhibits properties typical of canonical vertebrate FGFRs, including promotion of FGF activity, enhancement of neuritic outgrowth and signaling via MAPK and the transcription factor CREB. ApLRRTK also enhanced the synaptic efficiency of neurons known to mediate in vivo memory-related defensive behaviors. These data reveal a novel molecular regulator of neuronal function in invertebrates, provide the first evolutionary linkage between LRR proteins and FGFRs and unveil an unprecedented mechanism of FGFR gene diversification in primeval central nervous systems.

**Electronic supplementary material:**

The online version of this article (doi:10.1007/s00726-014-1803-2) contains supplementary material, which is available to authorized users.

## Introduction

Fibroblast Growth Factor Receptors (FGFRs) are cell-surface tyrosine kinase proteins that regulate a variety of essential biological functions, including embryological development, cellular growth, nervous system formation, adult neurogenesis and learning-related long-term synaptic plasticity (Itoh and Ornitz 2004; Itoh 2007; Coulier et al. 1997; Lessmann 1998; Reuss and von Bohlen und Halbach O 2003; Stevens et al. 2012; Zhao et al. 2007). Canonical FGFRs have been classically characterized by two major features: First, the distinctive presence of an extracellular region containing two to three Ig-like domains implicated in the interaction and signaling by Fibroblast Growth Factors (FGFs). And second, the presence of a highly conserved intracellular tyrosine kinase domain responsible for their phosphorylative catalytic activity (Eswarakumar et al. 2005; Coulier et al. 1997; Powers et al. 2000; Itoh and Ornitz 2004). We here studied ApLRRTK, a previously uncharacterized *Aplysia* receptor recently identified by Kassabov et al. (GenBank: FJ969839.1 and GenBank: ADB97918.1 (Kassabov et al. 2013)). We used a cutting age bioinformatical approach to learn about the biological identity of ApLRRTK and identified ApLRRTK as a member of the FGFRs gene family. Combined molecular biological and functional electrophysiological studies corroborated the bioinformatics predictions and unveiled ApLRRTK as an enhancer of FGF signaling and a promoter of neuronal outgrowth and memory-related synaptic plasticity. These data reveal a completely novel molecular regulator in invertebrate nervous systems. Moreover, we provide the first direct evolutionary link between LRR proteins and FGFRs, thus offering a novel molecular perspective for the evolutionary origins and diversification of FGFRs and LRR tyrosine kinase receptors.

## Results

### ApLRRTK is a member of the FGFRs gene family

**Fig. 1:**
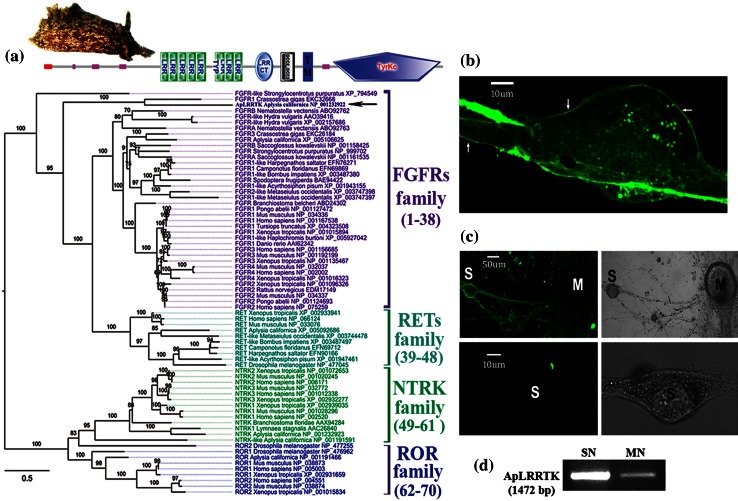
ApLRRTK is endogenously expressed in sensory and motor neurons of the *Aplysia* Gill-Withdrawal Reflex. **a** Above, a picture of an adult *Aplysia* californica. Below, domain composition (not scaled), of ApLRRTK; the extracellular domain of ApLRRTK contains eight LRR motifs and a GCC2_GCC3 region (*black* *and* *white* *box*) (Kassabov et al. 2013). A single-span transmembrane domain is defined between residues S922 and A944 (TM). The intracellular region contains a predicted tyrosine kinase domain (TyrKc) of 448aa (R945–T1393) significantly homologue to that of FGFRs (Supplementary Data, Supplementary Fig. 1). Domain composition predicted by SMART (Letunic et al. 2012; Schultz et al. 1998). Lower, maximum likelihood phylogenetic tree inferred from the multiple sequence alignments for the conserved tyrosine kinase domains of representative vertebrate and invertebrate FGFRs (in *purple*), ret proto-oncogene (RET in *cyan*), neurotrophic tyrosine kinase, receptor (NTRK in *green*), receptor tyrosine kinase-like orphan receptor (ROR in *blue*) and ApLRRTK (in *purple* with *arrow*) sequences (full names of all genes are provided in Supplementary Table 1). Midpoint rooting was used. Numbers along the branches of the tree show the IQ-TREE ultrafast bootstrap supports (“Materials and methods”). Scale bar represents the expected number of amino acid substitutions per alignment site. **b** ApLRRTK showed surface-membrane localization (*white arrows*) when overexpressed in sensory neurons. **c** Immunocytochemistry indicated membrane localization for ApLRRTK in sensory (*S*) and motor (*M*) co-cultured neurons of the *Aplysia* gill-withdrawal reflex (*upper left panel*). No signal was detected in negative control experiments (*lower left panel*). **d** RT-PCR analysis rendered a band with the expected size for ApLRRTK in both sensory (*SN*) and motor (*MN*) neurons. (Further details in Supplementary Data)

To determine the structural nature of ApLRRTK protein, we first performed bioinformatics analysis to predict its putative domain composition. The SMART (Letunic et al. 2012; Schultz et al. 1998) and CDART (Geer et al. 2002; Altschul et al. 1990) domain prediction algorithms indicated that ApLRRTK is a transmembrane protein with a unique structural composition: ApLRRTK contains an extracellular signal peptide followed by nine consecutive LRR motifs flanked by a GCC2_GCC3 region, a transmembrane domain and an intracellular tyrosine kinase, catalytic domain (Fig. 1a, upper panel). We then performed a phylogenetic analysis for the conserved tyrosine kinase domain of ApLRRTK, FGFRs and other vertebrate and invertebrate tyrosine kinase-containing proteins indentified via BLAST search (“Materials and methods”). The resulting phylogenetic tree (Fig. 1a; Supplementary Fig. 1 and Supplementary Table 1) revealed that ApLRRTK is a member of the FGFRs gene family.

### ApLRRTK is expressed in nervous system neurons

Immunohistochemical, immunocytochemical, single-cell RT-PCR and in situ hybridization analyses indicated that ApLRRTK is expressed in the *Aplysia* nervous system and localizes at the cell-surface plasma membrane (Fig. 1b–d, Supplementary Data and Supplementary Fig. 3), in agreement with the bioinformatics predictions (Fig. 1a). Overexpression experiments using an ApLRRTK plasmidic construct (in fusion with innocuous Green Fluorescent Protein (GFP)) substantiated localization of ApLRRTK to the plasma membrane of sensory neurons, matching the expression pattern of FGFRs (Fig. 1b).

### ApLRRTK promotes neuritic outgrowth and synaptic strengthening

**Fig. 2:**
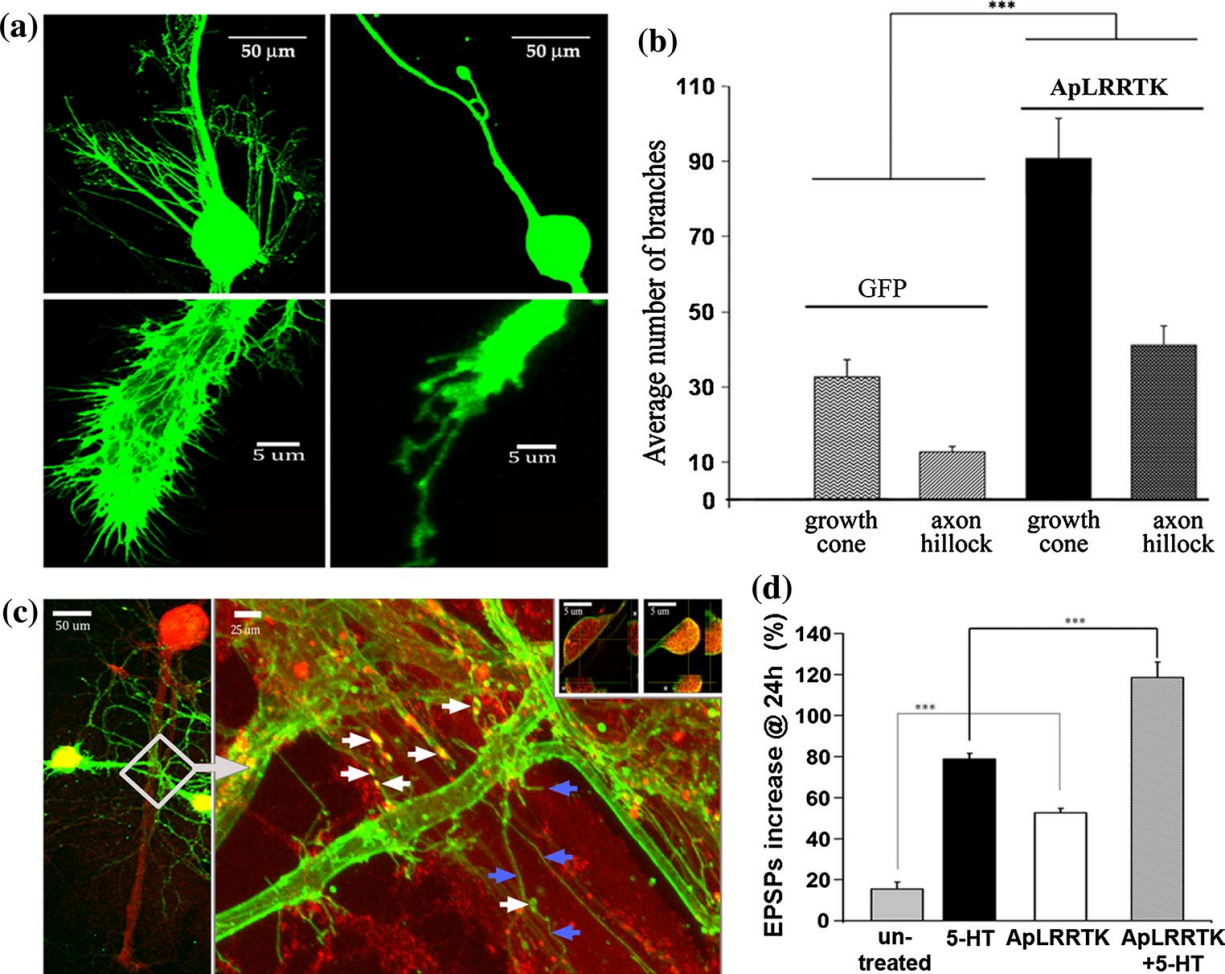
ApLRRTK promotes neuritogenesis, synaptic strengthening and 5-HT-induced plasticity. **a** ApLRRTK-GFP overexpression in presynaptic sensory neurons (*left panels*) promoted the outgrowth of elongated newly formed protrusive structures at the cell body, axon and growth cones when compared to control cells overexpressing GFP alone (*right panels*). **b** ApLRRTK overexpression induced significantly more protrusive structures (number of branches) than GFP-overexpressing control neurons (*n* = 6–8 per group). **c** Overexpression of ApLRRTK in presynaptic sensory neurons (*yellow–green*) making presumptive synaptic contacts with L7 motor neurons (*red*). Magnification (*white arrowed square*) shows the ApLRRTK-induced protrusive structures growing along the target motor neuron (*blue arrows*) and giving rise to *en-passant* varicose structures with the typical morphological properties of sensory neuron presynaptic contacts (*white arrows*). Insert (upper right), presynaptic structures examined in independent co-expression experiments contained both ApLRRTK (*green*) and Synaptophysin (*red*) further suggesting a role for ApLRRTK in synaptic functions (*asterisks* indicate lateral sections by the indicated cutting lines to better appreciate both ApLRRTK and Synaptophysin). **d** ApLRRTK enhanced basal synaptic strength and 5-HT-induced plasticity (*n* = 7–10 per group) as evaluated by the percentage of change in the amplitudes of EPSP (compared with initial EPSP amplitudes). Data presented as mean ± SEM ***p < 0.001

We next examined the effects of ApLRRTK overexpression on isolated neurons and on neurons with established functional neuronal circuits. In isolated sensory neurons, ApLRRTK induced the formation of long filopodial-like protrusions, some of which developed into thin neuritic projections (Fig. 2a). These filopodial structures were widely distributed among the cell body, axon and growth cones (Fig. 2a) and were morphologically distinct and quantitatively more pronounced compared to controls (Fig. 2a, b). Consistent with this observation, in in vitro reconstituted sensory-to-motor synaptic circuits, presynaptic overexpression of ApLRRTK also induced lengthened protrusions that projected towards the postsynaptic motor neuron and established physical contact. Many of these ApLRRTK-induced protrusions grew along the axon of the targeted motor neuron and developed *en-passant* varicose structures with the typical gross morphology of sensory neuron presynaptic contacts (Kandel 2001; Udo et al. 2005) (Fig. 2c). All ApLRRTK-induced protrusions remarkably resembled the filopodial-like neuritic outgrowth mediated by FGFRs (Li et al. 2011).

Given the robust morphological phenotype induced by ApLRRTK, we sought to determine whether this structural remodeling was also accompanied by functional electrical changes in the synaptic strength of the sensory-motor neuron synapses. We therefore electrophysiologically measured presynaptically evoked excitatory postsynaptic potentials (EPSPs) as an index of synaptic strength (Kandel 2001) before and 24 h after presynaptic overexpression of ApLRRTK. Overexpression of ApLRRTK resulted in a significant increase of the basal amplitude of the evoked postsynaptic electrical response as compared to control levels (Fig. 2d). Taken together, these observations suggest that ApLRRTK may act in vivo as a regulator of neuritogenesis and synaptic strengthening in the *Aplysia* central nervous system.

On the basis of the remarkable capability of ApLRRTK to promote synaptic strengthening by itself, we next explored whether ApLRRTK overexpression would attenuate or saturate the enhancement in synaptic strengthening typically induced by serotonin (5-HT) in sensory-motor neuron co-cultures (Kandel 2001). 5HT is a neurotransmitter that acts as a promoter of both neuritogenesis and synaptic strengthening and mediates the sensitization of the gill-withdrawal reflex in *Aplysia* (Kandel 2001). We found that ApLRRTK acted as an enhancer of 5-HT activity, as presynaptic overexpression of ApLRRTK significantly augmented the long-term synaptic strengthening typically induced after stimulation with five pulses of 5-HT (Fig. 2d).

### ApLRRTK shares conserved signaling features of FGFRs

**Fig. 3:**
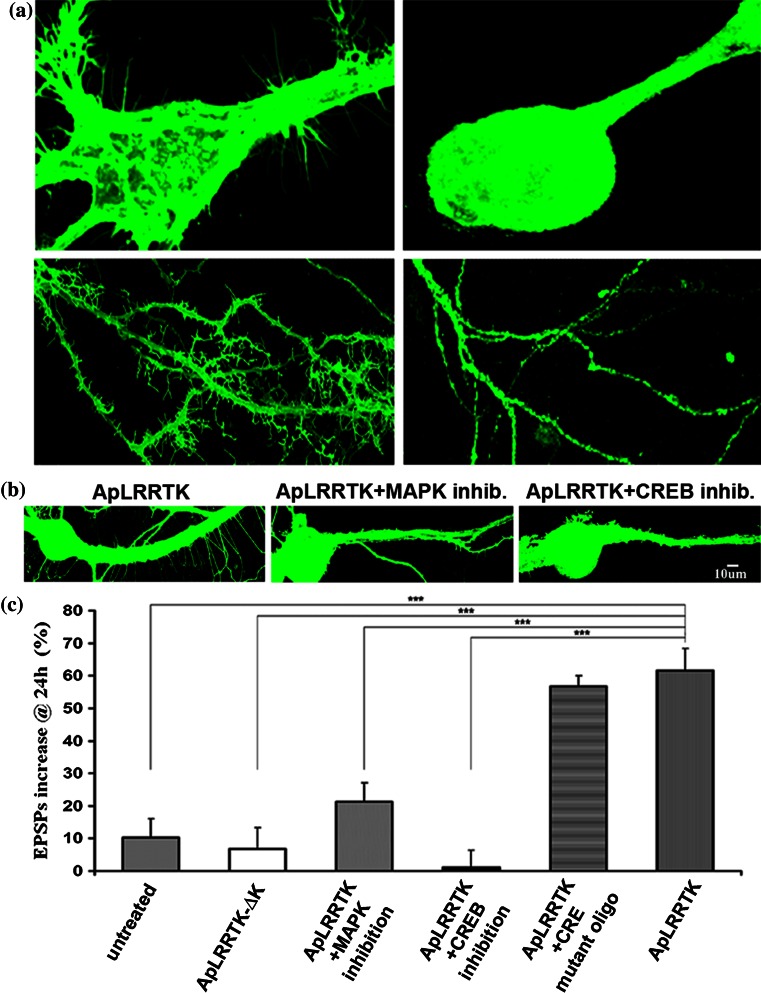
ApLRRTK function requires its tyrosine kinase domain, MAPK and CREB. **a** Presynaptic overexpression of ApLRRTK promoted neuritic outgrowth (*left upper* and *lower panels*). Conversely, deletion of ApLRRTK tyrosine kinase domain (ApLRRTK-ΔK deletion mutant) prevented ApLRRTK-induced neuritic outgrowth (*right upper* and *lower panels*). **b** Presynaptic inhibition of MAPK or CREB prevented the ApLRRTK-induced neuritic outgrowth. **c** Overexpression of ApLRRTK-ΔK and inhibition of MAPK or CREB impaired the synaptic strengthening induced by ApLRRTK, as evaluated by the percentage of change in the amplitudes of EPSP (compared with initial EPSP amplitudes). Overexpression of ApLRRTK in the presence of an injected CRE mutant oligo (Puthanveettil et al. 2008), solitary presynaptic overexpression of ApLRRTK and recordings from untreated co-cultures were used as controls (*n* = 8–10). All data are presented as mean ± SEM. ***p < 0.001

In FGFRs, the intracellular tyrosine kinase domain is critical for the mediation of the intracellular signaling cascades that underlie growth-related function (Reuss and von Bohlen und Halbach 2003; Eswarakumar et al. 2005). On the basis of the remarkable analogy observed between the tyrosine kinase domains of FGFRs and ApLRRTK (Supplementary Fig. 1), we next sought to evaluate the importance of the intracellular tyrosine kinase domain of ApLRRTK for the induction of neuritic outgrowth. To this end, we isolated sensory neurons and overexpressed a mutant form of ApLRRTK, lacking the intracellular kinase domain (ApLRRTK-ΔK, in fusion with innocuous GFP). 24 h later, we examined the effects of this overexpression on neuronal morphology. We found that the enhancement in neuritogenesis typically induced by ApLRRTK was abolished by the deletion of its kinase domain (Fig. 3a) indicating that ApLRRTK requires its intracellular tyrosine kinase domain to promote neuritic outgrowth. This result suggested that ApLRRTK could exert tyrosine kinase catalytic activity and that, consequently, ApLRRTK could potentially engage some of the same intracellular signaling cascades known to be regulated by FGFRs (Stachowiak et al. 2003; Tan et al. 1996). We tested this hypothesis using a non-radioactive tyrosine kinase activity assay (Supplementary Data) comparing transfected HEK cells expressing either the GFP protein alone, ApLRRTK-GFP or the ApLRRTK-ΔK-GFP deletion mutant. We found that expression of the full-length ApLRRTK-GFP led to a more than twofold increase in tyrosine kinase activity with respect to GFP alone, untransfected controls and HEK cells expressing the truncated ApLRRTK-ΔK-GFP lacking the kinase domain (untransfected controls = 1.3 ± 0.5 *n* = 4; GFP = 1.5 ± 0.6 *n* = 4; ApLRRTK-ΔK-GFP = 1.4 ± 0.3 *n* = 4; ApLRRTK-GFP = 3.8 ± 0.7 *n* = 4). We additionally explored the possibility that the actions exerted by ApLRRTK were related to the MAPK/CREB pathway, which is known to be involved in FGFRs signaling (Stachowiak et al. 2003; Tan et al. 1996). To this end, we first examined whether the observed promotion of neuritic outgrowth and the enhancement of synaptic strengthening induced by ApLRRTK depended on MAPK. Using a pharmacological inhibition protocol previously described for *Aplysia* neurons (Chin et al. 2006), we found that inhibition of MAPK almost entirely abolished the ApLRRTK-induced presynaptic neuritic outgrowth (Fig. 3b). These data are in agreement with previous observations relating the FGFs/FGFRs pathway to MAPK (Tan et al. 1996). Since the FGFs/FGFRs pathway acts through CREB signaling (Stachowiak et al. 2003; Tan et al. 1996), we examined whether CREB was required for the ApLRRTK-induced promotion of neuritic outgrowth and enhancement of synaptic strengthening. To this end, we injected a CRE element that binds phospho CREB resulting in CREB inhibition, a strategy that we have previously used for the inhibition of CREB in *Aplysia* neurons (Puthanveettil et al. 2008). We found that presynaptic ApLRRTK overexpression did not potentiate neuritic outgrowth in CREB-inhibition experiments (Fig. 3b). In addition, at the functional level, solitary overexpression of the mutant ApLRRTK-ΔK lacking a kinase domain did not potentiate synaptic strengthening and overexpression of the full-length ApLRRTK-GFP did not promote synaptic strengthening in MAPK or CREB inhibition experiments as examined electrophysiologically (Fig. 3c). These data are in agreement with previous observations relating the FGFs/FGFRs pathway to MAPK and CREB activity (Stachowiak et al. 2003; Tan et al. 1996) and collectively suggest that the predicted intracellular tyrosine kinase domain of ApLRRTK is active and necessary for ApLRRTK function, which requires the MAPK/CREB signaling pathway.

We moreover examined electrophysiologically the effects of recombinant FGFs on ApLRRTK overexpressed in sensory-motor synapses reconstituted in vitro and found that ApLRRTK enhanced the effects of FGF on synaptic strengthening (Supplementary Data; Supplementary Fig. 4b). These observations are in agreement with previous reports relating LRR proteins with the actions of FGFs (Bottcher et al. 2004; Maretto et al. 2008) (see also Supplementary Data). Further observations (Supplementary Data) also suggested that ApLRRTK might act in vivo as a neuronal receptor for putative *Aplysia* FGF-like peptides thus sharing most of the highly conserved signaling features typical of canonical vertebrate FGFRs.

### ApLRRTK promotes synaptic strengthening

**Fig. 4:**
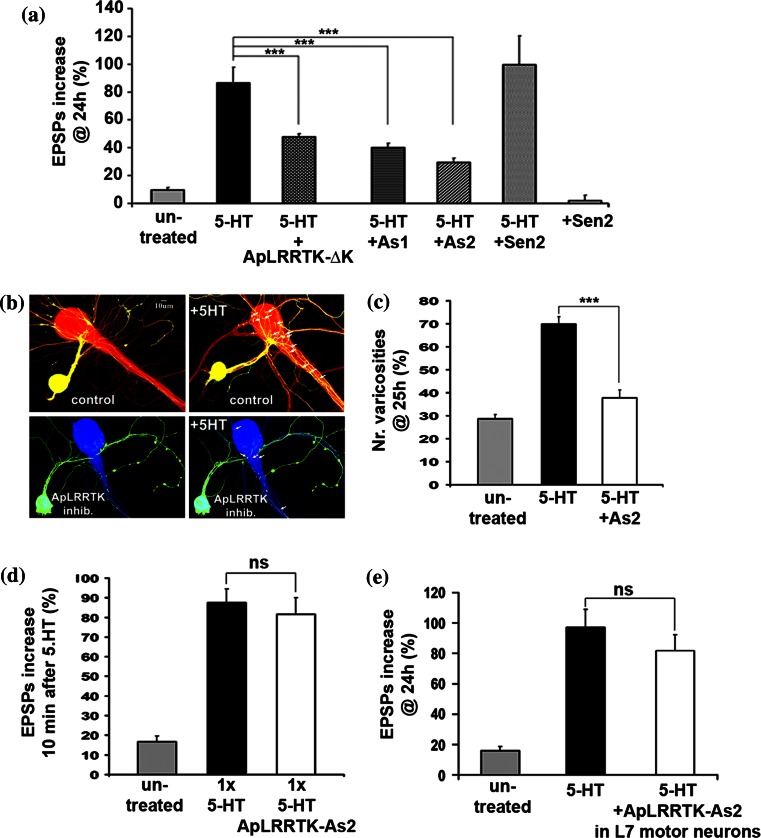
ApLRRTK selectively mediates presynaptic, long-term plasticity. **a** Presynaptically, both overexpression of ApLRRTK-ΔK and injection of ApLRRTK-specific antisense-oligonucleotides (*As1* and *As2*) impaired the increase in synaptic strength typically induced by five pulses of 5-HT (*n* = 10–18 per group) as evaluated by percentage changes in EPSP amplitudes (compared with initial EPSP amplitudes). **b** Presynaptic antisense-oligonucleotide inhibition of ApLRRTK reduced the number and extent of the 5-HT-induced outgrowth of presynaptic neuritic branches and the formation of new presynaptic varicosities. Upper left panel, a sensory neuron (*yellow*) contacts a postsynaptic motor neuron (*red*). The growth of new presynaptic branches and newly formed presumptive presynaptic contacts with L7 motor neurons is shown 24 h after five pulses of 5-HT (*white arrows*, *upper right panel*). Conversely, 24 h after ApLRRTK inhibition-treatment in presynaptic sensory neurons (*yellow-green*, *lower left panel*) results in a decreased number of presynaptic branches and presumptive synaptic contacts with L7 motor neurons (indicated by *white arrows*, *lower right panel*). **c** Presynaptic inhibition of ApLRRTK impaired the 5-HT-induced formation of new sensory neuron varicosities:  % of change in varicosity number (*n* = 10 per group). **d** Presynaptic inhibition of ApLRRTK using antisense-oligonucleotides (*As2*) did not prevent 5-HT-induced Short-Term Facilitation (STF) (*n* = 8–11 per group). **e** Postsynaptically, inhibition of ApLRRTK using antisense-oligonucleotides (*As2*) did not prevent 5-HT-induced Long-Term Facilitation (LTF) (*n* = 7–10 per group) as evaluated by percentage changes in EPSP amplitudes (compared with initial EPSP amplitudes). Data presented as mean ± SEM. ***p < 0.001, ns *p* > 0.05

In *Aplysia*, the neurotransmitter 5-HT mediates the sensitization of the gill-withdrawal reflex (Kandel 2001). This sensitization can be paradigmatically recapitulated by reconstituting in culture the monosynaptic component established between sensory and motors neuron that mediate the gill-withdrawal reflex in the intact animal (Kandel 2012). This co-culture has proven useful for the identification of molecular elements determining learning-related synaptic plasticity (Kandel 2001). In this model, 5-HT activates the MAPK/CREB pathway, which mediates the morphological changes underlying memory-related long-term synaptic plasticity during sensitization (Kandel 2001). Therefore, we used the co-culture to examine whether ApLRRTK mediated the 5-HT-induced long-term facilitation. We expressed the ApLRRTK-ΔK in presynaptic sensory neurons. ApLRRTK-ΔK acted as a negative modulator of the 5-HT-induced synaptic strengthening (Fig. 4a), suggesting that ApLRRTK is a downstream molecular player of 5-HT-induced synaptic strengthening. These data also suggested that ApLRRTK-ΔK interferes with the function of the endogenous ApLRRTK inducing the formation of dysfunctional, limping dimeric complexes resulting in less effective 5-HT-induced plasticity.

To substantiate these hypotheses, we examined the effects of ApLRRTK inhibition via intracellular microinjection of two different ApLRRTK-specific antisense-oligonucleotides (As1 and As2) following methods that we previously described (Puthanveettil et al. 2008). Inhibition of ApLRRTK hampered the typical 5-HT-induced long-term synaptic facilitation and increased number of presynaptic varicosities growing on the target motor neuron. No effects were observed in experiments injecting control sense-oligonucleotides (Fig. 4a–c).

We next examined whether ApLRRTK function was also required for the 5-HT-induced short-term facilitation, a paradigmatic model for short-term memory storage. We intracellularly microinjected ApLRRTK antisense-oligonucleotides into sensory neurons as described before (Puthanveettil et al. 2008); and 4 h later stimulated the sensory-motor co-cultures with a single 5 min pulse of 5-HT to induce short-term facilitation as previously established (Kandel 2001). We observed that short-term facilitation was not affected by depleting the levels of ApLRRTK following protocols previously described (Puthanveettil et al. 2008) (Fig. 4d).

We moreover examined whether the postsynaptic inhibition of ApLRRTK could also impair 5-HT-induced long-term synaptic plasticity. We found that postsynaptic microinjection of antisense-oligonucleotides specific for ApLRRTK did not impair the 5-HT-induced long-term facilitation (Fig. 4e), thus indicating that ApLRRTK has a selective presynaptic role.

## Discussion

FGFRs are known to promote structural presynaptic reorganization, neuronal outgrowth, and memory-related neuronal synaptic strengthening (Eswarakumar et al. 2005; Coulier et al. 1997; Powers et al. 2000; Itoh and Ornitz 2004; Reuss and von Bohlen und Halbach 2003; Stevens et al. 2012; Zhao et al. 2007). Here, we revealed ApLRRTK as the first known member of the FGFRs family that contains extracellular LRR motifs. In addition, we unveiled a role of ApLRRTK as an enhancer of ligand signaling and as promoter of presynaptic reorganization, neuronal outgrowth and behaviorally relevant synaptic strengthening.

The most striking results deriving from the bioinformatics analysis are the establishment of ApLRRTK as a phylogenetic member of the FGFRs gene family and the fact that the extracellular domain of ApLRRTK does not contain the distinctive peptide-binding Ig domains present in all canonical vertebrate and invertebrate FGFRs identified so far. The basic question emerging is whether absence of Ig motifs would preclude ApLRRTK of having peptide-binding properties. Although ApLRRTK has no Ig domains in its ectodomain, bioinformatics analysis indicates that ApLRRTK contains instead several extracellular LRR motifs. While Ig domains are prominently known for the ability to mediate the binding of signaling peptides, also LRR motifs have been described to act as adaptor molecules for the binding of distinct types of signaling ligand peptides in a variety of proteins (Kobe and Deisenhofer 1994; Buchanan and Gay 1996), including Tyrosine Kinase Receptors (Windisch et al. 1995a; Windisch et al. 1995b). For example, LRR motifs can be found in the extracellular domain of vertebrate TrkA and TrkB receptors (Schneider and Schweiger 1991; Windisch et al. 1995a; Windisch et al. 1995c) were they mediate the binding of associated neurotrophins with affinities in the nanomolar range (Windisch et al. 1995c). The functional relevance of ligand-binding LRR motifs in tyrosine kinase receptors is further supported by reports on the transcription of TrkB receptor genes into endogenous splice variants of TrkB in which some -or all- the LRR motifs are eliminated, resulting in responsive receptors, incapable of binding their related ligands (Ninkina et al. 1997). Moreover, in invertebrate central nervous systems, a previous study by van Kesteren et al. reported the existence of neurotrophin-binding receptors that belong to the vertebrate Trk gene family of Tyrosine Kinase Receptors and that although constitutively lack Ig motifs, they contain LRR domains associated to the binding of neurotrophins (van Kesteren et al. 1998). All these observations demonstrate that Ig domains are not a requisite for the binding of signaling peptides in LRR-containing Tyrosine Kinase Receptors.

**Fig. 5:**
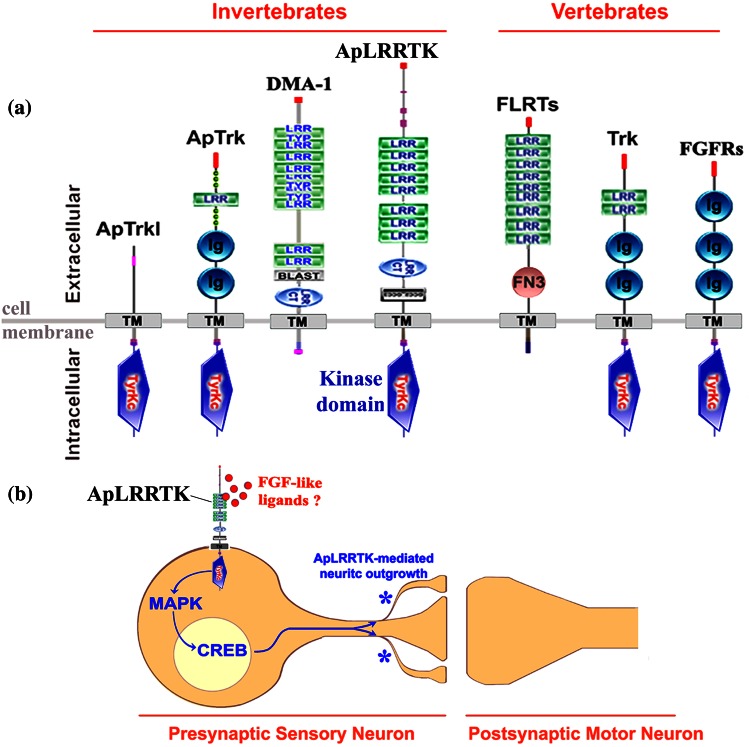
ApLRRTK and cell-surface vertebrates and invertebrates proteins. **a** Tyrosine kinase receptors and cell-surface non-kinase proteins from vertebrate and invertebrates can contain extracellular LRR domains, Ig-like motifs, or both. ApLRRTK is the first described tyrosine kinase receptor belonging to the FGFRs gene family whose ectodomain contains LRR motifs. Other proteins, either lack putative extracellular recognition motifs for the binding of signaling peptides as is the case of ApTrkl (Ormond et al. 2004), or if they do, they do not present an intracellular tyrosine kinase domain as for DMA-1 (Liu and Shen 2011) and FLRT proteins (Bottcher et al. 2004). Cell-surface tyrosine kinase receptors with extracellular LRR or Ig-like motifs can bind endogenous ligands or can be orphans (for whom no endogenous ligand has been yet identified but that can, however, bind exogenous signaling binding peptides). **b** Simplified functional model for the involvement of ApLRRTK in learning-related promotion of neuritic outgrowth and synaptic strengthening: A putative extracellular FGF-like signaling ligand (small *red circles*) binds to the LRR motifs of ApLRRTK triggering its tyrosine kinase activity in a manner analogue to that of FGFRs. Binding of the ligand could trigger ApLRRTK kinase activity thereby initiating several intracellular signaling cascades (*blue lines*) including MAPK and CREB activation, which are known to be important for long-term synaptic strengthening. MAPK-CREB, as it has been described (Kandel 2012), in turn promote presynaptic neuritogenesis and the formation of newly formed functional synaptic terminals (*blue asterisks*) resulting in a plastic strengthening of the neuronal circuit

The structural and molecular signaling commonalities between ApLRRTK and FGFRs, together with the capability of ApLRRTK to enhance the effect of FGFs, suggest the existence of endogenous FGF-like ligands in *Aplysia* that might act through ApLRRTK to regulate the morphological and functional properties of neurons in a manner analogue to that of classical FGFRs (Fig. 5). In fact, similarly to FGFRs, ApLRRTK contains an active intracellular tyrosine kinase domain with the conserved tyrosine residues shown to be involved in the phosphorylative activity of FGFRs (Stachowiak et al. 2003; Tan et al. 1996; Foehr et al. 2001). Also ApLRRTK signals via MAPK and CREB, both of which are implicated in long-term learning-related synaptic plasticity (Kandel 2001).

We hypothesize that the observed synaptic strengthening induced by ApLRRTK is mediated through the enhanced outgrowth of filopodial structures, which are known to allow the formation of synaptic contacts (Mattila and Lappalainen 2008) that are important for learning-related long-term synaptic strengthening (Cingolani and Goda 2008; Toni et al. 1999). In agreement with this hypothesis and supporting all the structural and functional commonalities between ApLRRTK and FGFRs, the promotion of filopodial growth and synaptogenesis induced by ApLRRTK in co-culture neurons resembles the presynaptic filopodial structures and synaptogenesis induced by FGFRs in co-cultured cells (Li et al. 2011). In a physiological context, ApLRRTK could also act as a plasticity-related inducible gene, since we further found that the levels of ApLRRTK mRNAs are increased in response to 5-HT (Supplementary Data and Supplementary Fig. 4f), which is in agreement with our previously published observations for neuronal proteins regulating synaptic functions (Puthanveettil et al. 2008).

Various models about the emergence of immunoglobulin-like domains of tyrosine kinase receptors have been proposed (Itoh and Ornitz 2004; Grassot et al. 2006). Similarly, different groups have addressed the evolutionary origins of FGFRs using both molecular and bioinformatics approaches (Agnes et al. 1997; Itoh and Ornitz 2004; Rebscher et al. 2009; Huang and Stern 2005; D’Aniello et al. 2008; Bertrand et al. 2014). However, in invertebrates, only few organisms have been described to have FGFRs (Coulier et al. 1997; Itoh and Ornitz 2004; Huang and Stern 2005; Agnes et al. 1997). For example, in the case of the *Aplysia* genus, despite abundant information on the genome, transcriptome (Moroz et al. 2006) and proteome (Monje et al. 2012), no canonical Ig-like-containing FGFRs have been identified thus far, to the best of our knowledge. Regulation of FGFs and FGFRs signaling by LRR proteins has proven to be important for several physiological processes, including embryogenesis, migration or axonal growth (Bottcher et al. 2004; Maretto et al. 2008; Morris et al. 2007; Skjerpen et al. 2002; Zhao et al. 2008; Zhen et al. 2012; Wang et al. 2003). Moreover, both LRR proteins and FGFs/FGFRs have been independently implicated in Alzheimer’s disease (Majercak et al. 2006; Tatebayashi et al. 1999), thereby highlighting the importance of clarifying the functional and evolutionary interrelationships between FGFs/FGFRs and LRR proteins. Previous studies have shown that diverse repertoires of immune-related receptors can be generated either through the rearrangement of immunoglobulin domains in some organisms, or exclusively through the recombinatorial assemblage of LRR modular units in other organisms lacking the immunoglobulin-based mechanism (Pancer and Cooper 2006). All these observations suggest the possibility that during evolution some primeval organisms might have become deprived of genes encoding for Ig-like containing FGFRs. Instead, those organisms might have diverged into alternative lineages in which transmembrane tyrosine kinase proteins belonging to the FGFRs gene family underwent the recombinatorial assemblage of genetic LRR modules resulting in the formation of FGFRs-family receptors serving functions analogue to those of canonical Ig-like containing FGFRs.

A large degree of attention has been given to Ig-like domains during the study of the evolutionary nature of tyrosine kinase receptors (Grassot et al. 2006; Rousset et al. 1995; Agnes et al. 1997; Itoh and Ornitz 2004; Itoh 2007; Coulier et al. 1997). Nevertheless, the importance of LRR in tyrosine kinase receptors evolution remains poorly investigated. Our work provides an unprecedented direct genetic linkage between LRR domains and FGFRs and highlights the biological relevance of LRR-containing receptors from the FGFRs gene family as critical regulators of neuronal function. These findings shed new light onto the evolutionary mechanisms of diversification of the FGFRs gene family based on the alternative presence of LRR motifs instead of Ig-like domains.

## Materials and methods

See Supplementary Data for image analysis of structural changes; In Situ Hybridization; Semi-Quantitative RT-PCR; HEK-293 Cell Culture and Transfection; Western Blotting; MAPK and CREB inhibition; Immunoprecipitation; Immunocytochemistry; Immunohistochemistry; Protein Tyrosine kinase Assay.

### Bioinformatics

We retrieved the tyrosine kinase-containing protein sequences (FGFR, NTRK, RET, and ROR) from the NCBI database. Sequences were identified by BLASTP searches using ApLRRTK (NP_001232922) and ApNTRK (NP_001232923) sequences as query. We downloaded the hidden markov model (HMM) for the TK domain from the Pfam database (Punta et al. 2012) and used hmmsearch3 (Eddy 2011) to extract the tyrosine kinase domains for all protein sequences. We then constructed the multiple amino acid sequence alignment for the tyrosine kinase domain sequences using MAFFT (Katoh and Standley 2013). Finally, we reconstructed the maximum likelihood phylogenetic tree and assessed clade supports using the IQ-TREE ultrafast bootstrap approximation (Minh et al. 2013). For tree reconstruction, we applied the LG + I + G + F substitution model as the best-fit model according to the Bayesian information criterion (Schwarz 1978). The resulting alignment and tree were visualized by Jalview (Waterhouse et al. 2009) and Figtree (http://tree.bio.ed.ac.uk/software/figtree/), respectively.

### Aplysia neuronal culture, protein expressions and electrophysiology

ApLRRTK-GFP and ApLRRTK-ΔK-GPF cDNA constructs cloned into the pNEX3 expression vector (Kaang 1996) were a generous gift from Dr. Stefan Kassabov. *Aplysia* sensory-motor neuron co-cultures, electrophysiological and antisense-oligonucleotide inhibition experiments were carried out as previously described (Kandel 2001; Puthanveettil et al. 2008).

### 5-HT treatment of sensory neurons

Sensory neuron containing pleural ganglia were isolated and connective tissue removed after 2 h of protease (Sigma, St. Louis, MO, U.S.A.) treatment. Ganglia were plated in L-15 culture media overnight to allow recovery from protease treatment. The next day, culture media was washed out and ganglia were treated with five pulses of 5-HT, as previously described (Monje et al. 2012). RNA was extracted 1 and 6 h after 5-HT treatment and RT-PCR analysis was carried out as described above.

### Statistical analysis

Differences between groups were analyzed by one-way ANOVA tests followed by Tukey–Kramer Multiple Comparisons Tests. An α-level of 0.05 was adopted in all instances. All analyses were carried out using BioStat 2009 professional software (AnalystSoft Inc).

## Electronic supplementary material

Below is the link to the electronic supplementary material.

Supplementary material 1 (DOC 156 kb)

Supplementary material 2 (TIFF 4687 kb)

Supplementary material 3 (TIFF 946 kb)

Supplementary material 4 (TIFF 1726 kb)

Supplementary material 5 (TIFF 876 kb)

Supplementary material 6 (TIFF 3434 kb)

Supplementary material 7 (DOC 36 kb)

## Abbreviations

LRR: Leucine-Rich Repeats
FGFs: Fibroblast Growth Factors
FGFRs: Fibroblast Growth Factor Receptors
Ig: Immunoglobulin
