# Supplementary material for: A novel Fibroblast Growth Factor Receptor family member promotes neuronal outgrowth and synaptic plasticity in Aplysia
Source: Amino Acids. 2014 Jul 25;46(11):2477–88. doi: 10.1007/s00726-014-1803-2 (PMC4200351; doi:10.1007/s00726-014-1803-2)
Supplement: Supplementary file 1 — Supplementary material 1 (DOC 156 kb) [file 726_2014_1803_MOESM1_ESM.doc]

**A novel Fibroblast Growth Factor Receptor family member promotes neuronal outgrowth and synaptic plasticity in *Aplysia***

**Daniela D. Pollak^1^, Bui Quang Minh^2^, Ana Cicvaric^1^ and Francisco J. Monje^1*^**

*^1^ Department of Neurophysiology and Neuropharmacology, Medical University of Vienna, Schwarzspanierstrasse 17, 1090 Vienna, Austria*

*^2^Center for Integrative Bioinformatics Vienna, Max F Perutz Laboratories, University of Vienna, Medical University of Vienna, Vienna, Austria.*

^*^Correspondence to:

[francisco.monje@meduniwien.ac.at](mailto:francisco.monje@meduniwien.ac.at)

**SUPPLEMENTARY DATA**

**Primer sequences used in *Semi-Quantitative RTPCR***

P1-F1: 5’ ATGAATGCCATTAGAATCTACAGCATTTACGCGTGC 3’ and

P1-R1: 5’ ACGAACATTGCCCATTGTCTGGGGCCTGCT 3’;

P2-F1: 5’ GGAATACGCTGAGCTGGGTGACCTCTTGAGCTACCT 3’ and

P2-R1: 5’ GTTCTCATGATGCGATACAGTTGCTCCGGGCAGGCT 3’;

GAPDH-RT-F1: 5’ CCGTATTGGTCGCCTGACTCTTCGT 3’ and

GAPDH-RT-R1: 5’TGACCACCTGCAGGTCCTTGGTGT 3’.

RT PCR for Figure 2.G was performed with the primer pair CTestUp2 and Low5:

GTCCTGGACCATCTGCATCTCCGGAGTATCAC - CTestUp2

GGACAAGGGAGGGCATGCGTGACAACC - Low5

***Antisera***

Affinity purified rabbit polyclonal antibodies raised against the ApLRRTK peptide sequence IDFNFRPSLPKDLDSPFFRV were a gift from Dr. Eric Kandel. These antibodies were used in immunoprecipitation experiments to enrich the ApLRRTK protein for masspectrometrical analysis to further confirm the specificity of the protein bands recognized (see below). Although suited for immunoblotting, these antibodies could not be successfully used for immunoprecipitation. To circumvent this problem, we used protein extracts of HEK-cells overexpressing ApLRRTK-GFP and verified that the band detected by our polyclonal ApLRRTK antibody corresponded in size (circa 180 kDa) to the band detected by a commercially available GFP-antibody (Supplementary Figure 2*d*). When the GFP-antibody was used for immunoprecipitation a single band at the expected molecular weight of APLRRTK-GFP was revealed by silver staining (Supplementary Figure 2*e*). This band was then used for masspectrometrical analysis by ESI-LC-MS/MS. Peptide mass fingerprints from this band led to the identification of fourteen peptides forming part of the ApLRRTK protein sequence, thus further confirming the specificity of the ApLRRTK antibody (Supplementary Figure 5). While the predicted molecular weight of ApLRRTK is ~150kDa (Kassabov et al. 2013), western blot experiments with protein extracts from *Aplysia* ganglia showed the presence of two bands, one of 95 kDa and other of about 300 kDa (Supplementary Figure 2*b*). The presence of a higher molecular weight band detected after incubation with specific antibodies seem to be a typical characteristic of cell-surface Tyrosine Kinase Receptors most likely forming dimeric complexes as it has been previously reported for neuronal *Aplysia* Tyrosine Kinase Receptors (Kassabov et al. 2013) and as is also typical of Fibroblast Growth Factor Receptors (Nur et al. 2008). These observations therefore suggest that ApLRRTK might be stabilized in a SDS-resistant covalent bond complex between the receptor itself, linear polysaccharides, or potential endogenous ligands present in the extracellular matrix. Although ApLRRTK could form dimeric or multimeric complexes, it also has several predicted target residues for glycosylation, which could favor the binding to proteoglycans or putative ligand molecules and result in molecular weights higher than those theoretically predicted, as also shown for some Fibroblast Growth Factor Receptors (Duchesne et al. 2006). The small fragment might represent a truncated form of ApLRRTK, since structurally-related Fibroblast Growth Factor Receptors can undergo rapid proteolytic cleavage soon after synthesis of full-length receptors, hereby giving rise to truncated transmembrane fragments (Hanneken et al. 1995; Hanneken et al. 1994; Pandit et al. 2002). ApLRRTK also shares major structural features with the members of the non-kinase cell-surface Fibronectin-Leucine-Rich transmembrane (FLRT) family of proteins (Bottcher et al. 2004), which can modulate FGF signaling and directly, physically interact with FGFRs to modulate its function. Interestingly, the extracellular domains of FLRT proteins are known to be subject of cleavage by metalloproteases giving rise to soluble fractions of the proteins that have functional properties (Yamagishi et al. 2011) thus suggesting the possibility that also ApLRRTK or its dimeric or multimeric complexes might be functionally fractionated into fragments of different size by the activity of metalloproteases.

***Image Analysis of Structural Changes***

Analysis of neuronal structural changes was performed by conventional confocal microscopy (Fluoview FV1000, Olympus, Center Valley, PA, U.S.A.). To estimate the number of sensory neuron varicosities we counted the total of labeled axonal swellings with a mean diameter greater than 3 µM in length and that appear in contact with the cell body and the initial segment of the postsynaptic motor neurons (labeled with Alexa whole-cell markers; Molecular Probes, Invitrogen Grand Island, NY, U.S.A.) as previously described (Bailey et al. 1992).

***In Situ Hybridization***

Two 500 bp fragments (one from the ORF corresponding to extracellular domain and another corresponding to the intracellular domain) from the ApLRRTK cDNA were cloned into the PCR TOPO II vector, linearized with Hind III and transcribed with T7 RNA polymerase (Roche, Indianapolis, U.S.A.) in the presence of digoxigenin (DIG) RNA labeling mix to make ApLRRTK antisense riboprobes (5’ GTTGCGAGCAGCAAGATCTCTGTGGAT 3’). For the sense probes (5’ ATC CACAGAGATCTTGCTGCTCGCAAC 3’) the plasmids were linearized with Xba-I and transcribed with SP6 RNA polymerase. After DNAse I treatment, the sense and antisense ribo-probes were used for *in situ* hybridization. About 1 ng of labeled RNA per μl of hybridization solution was used per culture dish. Sensory neuron-motor neuron cultures (4-5 days old) were washed with artificial seawater and fixed for 10 minutes at room temperature with 2 ml of 4% paraformaldehyde in artificial seawater and washed three times in PBS. The *in situ* hybridization and detection of mRNAs using a Fluorescent Antibody Enhancer kit (Roche, Indianapolis, U.S.A.) for DIG detection were followed as previously described (Puthanveettil et al. 2008). Images were acquired using a Fluoview 1000 confocal microscope (Olympus, Center Valley, PA, U.S.A.) with a 20x objective.

***Semi-Quantitative RTPCR***

Total RNA was isolated using Trizol reagent (Invitrogen Grand Island, NY, U.S.A.) and 500 ng of total RNA were used to perform semi-quantitative RTPCR (one step RTPCR kit-TakaRa Bio Inc). ApGAPDH was used as endogenous control. After 25 cycles of PCR, the products were run on a 2% agarose gel. Primer sequences are available on demand.

***HEK-293 Cell Culture and Transfection***

Cells were maintained in DMEM (Sigma Aldrich St. Louis, MO, U.S.A.) supplemented with 10% fetal bovine serum (Sigma Aldrich St. Louis, MO, U.S.A.) and antibiotics until cells reached 80–90% confluence. The day before transfection, cells were seeded at 80% confluence in 35 mm dishes. Cells were transfected using either Lipofectamine 2000 (Invitrogen Grand Island, NY, U.S.A.) or Fugene (Roche, Indianapolis, U.S.A.) following the manufacturers' protocols. 24 hrs post-transfection, cells were washed in pre-chilled PBS and collected in 0.5 ml of lysis buffer and used for further analyses.

***Western Blotting***

*Aplysia* ganglia tissue was homogenized and proteins solubilized in a buffer containing 8M Urea, 2% SDS, protease inhibitor cocktail (Roche, Indianapolis, U.S.A.) and 1mM PMSF. The protein content was determined by the BCA method (Pierce Biotechnology-Thermo Scientific, Rockford, IL U.S.A.). Samples (25 μg protein) were re-suspended in 2xLaemmli sample buffer (Bio-Rad, Hercules, CA, U.S.A.) loaded onto SDS-PAGE gels and run at constant current (40mA per gel). Proteins separated on the gel were transferred onto PVDF membranes (Millipore, MA, USA). Membranes were blocked for one hour in PBS containing 5% non-fat milk (Carnation, Markham, ON, Canada) and incubated with primary antibodies diluted in blocking solution overnight at 4◦C (anti-ApLRRTK 1:2000, anti-GFP (Invitrogen Grand Island, NY, U.S.A.) 1:1000) and detected with horseradish peroxidase coupled secondary antibodies 1:5000 (Jackson ImmunoResearch). Immunoblotting was also carried out in the absence of primary antibodies. Membranes were developed with the ECL Plus™ chemiluminescence reagent (General Electric Healthcare, Fairfield, Connecticut, U.S.A.). Apparent molecular masses were determined by running standard protein markers (Bio-Rad, Hercules, CA, U.S.A.) ranging from 10 to 250 kDa.

***MAPK and CREB inhibition***

The inhibition of MAPK protein activity was carried out using U0126 (20 µM in 0.2% DMSO, Promega) as previously reported for *Aplysia* neurons (Chin et al. 2006). 6 hours after injection of the ApLRRTK DNA construct, cells were treated for 4.5 h with U0126. Long-term effects of MAPK inhibition on ApLRRTK overexpressing cells were examined 24 hr after ApLRRTK DNA injection. CREB inhibition experiments were performed as previously described (Puthanveettil et al. 2008).

***Immunoprecipitation***

HEK cells transfected with ApLRRTK-GFP or GFP alone (controls) were homogenized in homogenization buffer (50 mM Tris, 150 mM NaCl, 0.5% NP40, 0.5% Triton, 0.5% sodium deoxychloate, protease inhibitor cocktail (Roche, Indianapolis, U.S.A.) and 1mM PMSF. The protein content of the supernatant was determined by the BCA method (Pierce Biotechnology-Thermo Scientific, Rockford, IL U.S.A.) and 300ug of protein was used for each immunoprecipitation. Samples were pre-cleared for 1 hour in 50ul of a 1:1 protein-A sepharose bead slurry (Sigma Aldrich St. Louis, MO, U.S.A.). Beads were spun down and the supernatant was incubated with 5 ug of antibody overnight at 4C. The next day 50ul of a 1:1 protein-A sepharose bead slurry (Sigma Aldrich St. Louis, MO, U.S.A.) was added and samples were incubated for 2 hours at 4C. Beads were then spun down and washed three times with homogenization buffer. Finally, samples were boiled with Laemmli buffer (Bio-Rad, Hercules, CA, U.S.A.) and run on SDS-PAGE gels. Gels were then either silver stained (Silver Quest Invitrogen Grand Island, NY, U.S.A.) or used for Western blot experiments.

***Immunocytochemistry***

Cells were washed with artificial sea water and fixed in 4% PFA plus 30% sucrose for 30 min. Cells were then incubated in permeabilization solution (0.1% Triton X-100, 30% sucrose, 1X PBS) for 10 min at RT. Samples were incubated in blocking solution (10% fetal bovine serum, 0.1% Triton X-100 in 1X PBS) for 1 hour, followed by incubation in primary antibody in blocking solution o/n at 4C, three washes with 1XPBS, secondary antibody in blocking solution incubation for 1 h and three more washes in 1X PBS. Antibody dilutions were 1:100 for primary antibodies, and 1:1000 for fluorescence- coupled secondary antibody (Molecular Probes, Invitrogen Grand Island, NY, U.S.A.). Confocal images were acquired using a Fluoview FV1000 microscope (Olympus, Center Valley, PA, U.S.A.).

***Immunohistochemistry***

*Aplysia* pleural ganglia were fixed in 4% PFA, 25% Sucrose in NaH2PO4 at pH 7.6 overnight at 4C. The following day, 18µm thick sections were cut on a cryostat and mounted on SuperFrost Plus slides (Fisher, Thermo Fisher Scientific Inc.Waltham, Massachusetts, U.S.A.). Slides were air-dried for 1 hour and then washed in 1xPBS for 5 min, followed by three washes (5min each) in 1XPBS + 0.3 % Triton Tx-100 (Sigma Aldrich St. Louis, MO, U.S.A.). Slides were then blocked for 1hr at RT in 10% fetal bovine serum in 1XPBS and incubated with a primary antibody (1:100) in a humid chamber overnight at 4◦C. After 3 washes (5 min each) in 1XPBS + 0.3 % Triton Tx-100, sections were incubated with a fluorescence- coupled secondary antibody (1:1000) (Molecular Probes, Invitrogen Grand Island, NY, U.S.A.) in a humid chamber 1hr at RT, washed 3 times (5 min each) in 1XPBS + 0.3 % Triton Tx-100, air dried, mounted with Fluosave (Calbiochem, Merck KGaA, Darmstadt, Germany) and coverslipped. Nuclear stainings were carried out using the DAPI marker.

***Protein Tyrosine-kinase Assay***

Cells were homogenized in a lysis buffer containing 50 mM HEPES pH 7.4, 0.5% Triton X-100, 10% glycerol, 1 mM dithiothreitol (DTT), 1 mM sodium vanadate and protease inhibitor cocktail (Roche, Indianapolis, U.S.A.) on ice. The protein content of the supernatant was determined by the BCA method (Pierce Biotechnology-Thermo Scientific, Rockford, IL U.S.A.) and equal amounts of protein were used in each reaction. The protein tyrosine-kinase assay was applied following the supplier’s protocol (Sigma Aldrich St. Louis, MO, U.S.A.). In brief, the substrate of the test, containing glutamic acid and multiple tyrosine residues was phosphorylated by EGFR (control) or test samples. The phosphorylated substrate was then visualized by an antibody coupled to HRP and a color reaction mediated by HRP was quantified by spectrophotometry and reflects the relative amount of tyrosine-kinase activity in the sample. 3-fold replicates of a blank control (no EGFR or test sample), different dilutions of EGFR and the test samples extracts were run on one microtiter plate. Protein Tyrosine-kinase activity in the sample was extrapolated from the EGFR standard curve (absorbance at 492 nm vs. units of EGFR activity).

**ApLRRTK is expressed in the Central Nervous system**

We used RT-PCR to evaluate the expression of ApLRRTK in different organs of *Aplysia* and found that ApLRRTK is highly expressed in the nervous system, not detectable in samples of muscle and ovotestis, and weakly present in the heart (Supplementary Figure 2). To determine the presence and distribution of the protein ApLRRTK in the nervous system we used polyclonal antibodies (a generous gift from Dr. Eric Kandel) targeting a specific sequence located in the extracellular portion of ApLRRTK.

Immunocytochemistry analysis depicted in Figure 1*c* was conducted using sensory and motor neurons of the *Aplysia* gill-withdrawal reflex that had been co-cultured *in vitro*. This sensory-to-motor neuron co-culture has been widely studied in the context of learning and memory as it paradigmatically mimics several of the major structural and functional changes that underlie the sensory-to-motor neuron synapses *in vivo* during the memory-related strengthening of defensive behaviors (Kandel 2001). Immunocytochemistry analysis revealed ApLRRTK to be localized to the plasma membrane of both presynaptic sensory neurons and postsynaptic motor neurons (Figure 1*c*). We also verified the expression profile of ApLRRTK in neurons from a behaviorally-relevant circuit by performing single-cell RT-PCR using isolated sensory and motor neurons known to mediate *in vivo* the gill-withdrawal reflex in *Aplysia* (Kandel 2001). RT-PCR results indicated that ApLRRTK is present in both sensory and motor neurons (Figure 1*d*). To further substantiate the RT-PCR results we used *in situ* hybridization in order to explore for the presence of neuronal ApLRRTK mRNAs as we have previously described in *Aplysia* cells (Puthanveettil et al. 2008). *In situ* hybridization assay allowed the detection of signals for ApLRRTK in both sensory and motor neurons (Supplementary Figure 3*b*). These observations indicate that ApLRRTK is a transmembrane protein (as also predicted by bioinformatics) primarily expressed in the *Aplysia* nervous system and present in both sensory and motor neurons of the *Aplysia* gill-withdrawal reflex (Kandel 2001).

**ApLRRTK mRNA levels are regulated by serotonin**

Depending upon the number of repetitions, stimulation with 5-HT can induce either short-term or long-term forms of learning-related synaptic potentiation of the *Aplysia* sensory-motor synapses (Kandel 2001). The observed selective involvement of ApLRRTK in long-term 5-HT-induced synaptic plasticity therefore raised the intriguing possibility that transcription of ApLRRTK itself could be subjected to regulation by 5-HT, hereby mediating some of the molecular events triggered by 5-HT and leading to long-term synaptic strengthening. To explore this possibility, we examined the effects of long-term facilitation-inducing repetitive stimulation with 5-HT (Kandel 2001) on the mRNA levels of ApLRRTK by RT-PCR. A significant increase in ApLRRTK mRNA levels detectable starting at 1 hour and lasting at least 6 hours after 5-HT stimulation was observed (Supplementary Figure 4*f*).

**ApLRRTK cross-react with FGF and promotes FGF-induced synaptic strengthening**

Based on previously reported observations that LRR proteins can modulate the FGFs/FGFRs signaling cascade (Bottcher et al. 2004; Maretto et al. 2008; Morris et al. 2007; Skjerpen et al. 2002; Zhao et al. 2008; Zhen et al. 2012; Wang et al. 2003) and considering the significant structural homology between ApLRRTK and FGFRs, we next examined the effects of the application of recombinant FGFs in ApLRRTK overexpression experiments using learning-related sensory-motor synapses reconstituted *in vitro*. In an electrophysiological screening assay, we first evaluated the results of bath application of different FGFs (FGF3, FGF9 and FGF4) on the synaptic strength of previously formed sensory-motor functional synapses. We found only FGF4 to exert a moderate strengthening of the synapses (Supplementary Figure 4*a*), consistent with previous reports in other animal model systems (Miyagawa et al. 1993; Abe et al. 1992; Szebenyi et al. 2001). FGF4 has been previously shown to promote neuronal differentiation in cell cultures (Kosaka et al. 2006). We therefore next set-out to examine a possible functional link between FGF4 and ApLRRTK by analyzing the synaptic effects of FGF4 on sensory-motor co-cultures in which ApLRRTK was overexpressed presynaptically. We found a pronounced ApLRRTK-mediated synergistic enhancement of synaptic strengthening after FGF4 stimulation (Supplementary Figure 4*b*). These results are in agreement with previous reports relating LRR proteins with the actions of FGFs (Bottcher et al. 2004; Maretto et al. 2008; Morris et al. 2007; Skjerpen et al. 2002; Zhao et al. 2008; Zhen et al. 2012; Wang et al. 2003).

To examine whether the application of the recombinant FGF4 could mimic the activity of a related *Aplysia* molecule, we tested whether, as previously shown for other proteins (Monje et al. 2012; Monje et al. 2013; Shaw et al. 2004; Si et al. 2010; Moreno et al. 1998; van Kesteren et al. 1998), an antibody directed against mammalian FGF4 could cross-reactively detect a presumed similar protein in *Aplysia*. Indeed, in Western Blot experiments using protein extracts from *Aplysia* neuronal tissue, a single band comparable to that observed using mouse brain tissue protein extracts, was detected at the molecular weight expected for FGF4 (20-30 kDa) (Supplementary Figure 4*c*) whereas no band was detected when an antibody raised against mammalian FGF1 was used (Supplementary Figure 4*d*). These observations suggest the possible existence of endogenous *Aplysia* FGF-like peptides that could potentially interact with ApLRRTK. Moreover, co-immuno precipitation experiments also provided first available evidence for a possible direct interaction between ApLRRTK and putative FGF-like endogenous peptides from *Aplysia* (Supplementary Figure 4*e*).

**Supplementary Data Figure Legends**

**Supplementary Figure 1**. Multiple amino acid sequence alignment has been constructed by MAFFT for the conserved Tyrosine Kinase domains of representative vertebrate and invertebrate FGFRs and ApLRRTK proteins. The most highly conserved amino acid residues are indicated in Navy blue. Receptor name, specie and accession number are also indicated.

**Supplementary Figure 2**. ***a)*** RT-PCR indicates a high expression of ApLRRTK in the CNS and a weak presence in the heart. No detectable signals were found in muscle and ovotestis. ***b)*** Two bands (around 95 and 290 kDa) were detected by polyclonal ApLRRTK antibodies in Western blot experiments using Aplysia ganglia protein extracts. ***c)*** Peptide competition assay renders no detectable signal in membranes co-incubated with the corresponding blocking peptide and no signal was also detected when the primary antibody was omitted, indicating the specificity of the antibody. ***d)*** Western blotting using protein extracts of HEK-cells overexpressing ApLRRTK-GFP. The ApLRRTK polyclonal antibody detected a band corresponding in size (circa 180 kDa) to the one detected by a commercially available GFP-antibody. ***e)*** A single and strong band at the expected molecular weight of ApLRRTK-GFP was visualized by silver staining using a GFP-antibody for immunoprecipitation. Mass spectrometric analysis by ESI-LC-MS/MS corroborated the identity of this band as containing the corresponding ApLRRTK sequence fragment (Supplementary Figure 5).

**Supplementary Figure 3**. ***a)*** Immunohistochemical analysis of Aplysia pleural ganglia sections using the ApLRRTK antibody (upper panel) reveals localization of ApLRRTK (red) at the neuronal external plasma membrane. DAPI as nuclear marker is displayed blue. No detectable membrane signal was observed in control experiments omitting the primary antibody (lower panel). ***b)*** In situ hybridization experiments indicate the presence of ApLRRTK mRNA in sensory (S) and motor (M) neurons, whereas no signal is detected in control (untreated and sense) experiments.

**Supplementary Figure 4. ApLRRTK interacts with -and enhances the effect of- FGFs**

***a)*** Recombinant FGF4 (mouse) induced a significant synaptic strengthening in *Aplysia* sensory-motor synapses reconstituted *in vitro* (n=10-14 per group) as evaluated by percentage changes in EPSP amplitudes (compared with initial EPSP amplitudes). ***b)*** This FGF4-induced synaptic strengthening was markedly augmented when in neurons overexpressing ApLRRTK. ***c)*** A band at the predicted molecular weight of FGF4 was detected in Western blots experiments using a FGF4 antibody (mouse) and employing protein extracts from *Aplysia-*ganglia and mouse whole brain tissue. ***d)*** No signal in protein extracts from *Aplysia-*ganglia was detected when a mouse FGF-1 antibody was used. ***e)*** A signal for FGF4 was detected in Western Blot experiments after immunoprecipitation using an ApLRRTK antibody only in protein extracts from HEK cells expressing ApLRRTK-GFP but not GFP alone. Data presented as SEM *** p < 0.001. ***f)*** Treatment with 5-HT resulted in a significant increase in the ApLRRTK mRNAs levels after 1 and 6 hours as evaluated by RT-PCR. mRNA levels of Sensorin are used as endogenous control. Data presented as mean ± SEM. ** p < 0.01, *** p < 0.001, ns p>0.05.

**Supplementary Figure 5.** ***Mass Spectrometry.*** *In-gel digestion of APLRRTK -containing gel bands:* Each gel band was transferred to a clean tube and broken up with the tip of tweezers. The protein was reduced by adding 100ul 0.01M DTT/0.1M Tris, pH 8.5, and heating at 55° for 1-2h. After cooling the tube to room temperature, the liquid was removed and replaced with 0.015M iodoacetamide/0.1M Tris, pH 8.5. This was allowed to react for 30 min. in the dark after which the liquid was removed and the gel was washed once with 200ul 0.05M Tris, pH 8.5/ 25% acetonitrile and twice with 200 ul 0.05M Tris, pH 8.5/50% acetonitrile for 20 min. with shaking. After removing the washes, the gel was dried for 30 min. in a Speed-Vac concentrator and rehydrated by adding 0.08 ug trypsin (sequencing grade, Roche, Indianapolis, U.S.A.) in 25ul 0.025M Tris, pH 8.5. The tube was placed in a heating block at 32° and left overnight. Peptides were extracted with 2x 50 ul 50% acetonitrile/2% TFA. The combined extracts were reduced in volume to ~20ul and transferred to an injection vial. ESI-LC-MS/MS analysis was done on a Micromass Q-Tof hybrid quadrupole/time-of-flight mass spectrometer with a nanoelectrospray source. Capillary voltage was set at 1.8kV and cone voltage 32V; collision energy was set according to mass and charge of the ion, from 14eV to 50eV. Chromatography was performed on an LC Packings HPLC with a C18 PepMap column using a linear acetonitrile gradient with flow rate of 200 nl/ min. Raw data files were processed using the MassLynx ProteinLynx software with the MaxEnt algorithm.

**References**

Abe K, Ishiyama J, Saito H (1992) Effects of epidermal growth factor and basic fibroblast growth factor on generation of long-term potentiation in the dentate gyrus of fimbria-fornix-lesioned rats. Brain Res 593 (2):335-338. doi:0006-8993(92)91332-9 [pii]

Bailey CH, Chen M, Keller F, Kandel ER (1992) Serotonin-mediated endocytosis of apCAM: an early step of learning-related synaptic growth in Aplysia. Science (New York, NY 256 (5057):645-649

Bottcher RT, Pollet N, Delius H, Niehrs C (2004) The transmembrane protein XFLRT3 forms a complex with FGF receptors and promotes FGF signalling. Nature cell biology 6 (1):38-44. doi:10.1038/ncb1082

ncb1082 [pii]

Chin J, Liu RY, Cleary LJ, Eskin A, Byrne JH (2006) TGF-beta1-induced long-term changes in neuronal excitability in aplysia sensory neurons depend on MAPK. Journal of neurophysiology 95 (5):3286-3290

Duchesne L, Tissot B, Rudd TR, Dell A, Fernig DG (2006) N-glycosylation of fibroblast growth factor receptor 1 regulates ligand and heparan sulfate co-receptor binding. The Journal of biological chemistry 281 (37):27178-27189. doi:M601248200 [pii]

10.1074/jbc.M601248200

Hanneken A, Maher PA, Baird A (1995) High affinity immunoreactive FGF receptors in the extracellular matrix of vascular endothelial cells--implications for the modulation of FGF-2. J Cell Biol 128 (6):1221-1228

Hanneken A, Ying W, Ling N, Baird A (1994) Identification of soluble forms of the fibroblast growth factor receptor in blood. Proceedings of the National Academy of Sciences of the United States of America 91 (19):9170-9174

Kandel ER (2001) The molecular biology of memory storage: a dialogue between genes and synapses. Science (New York, NY 294 (5544):1030-1038

Kassabov SR, Choi YB, Karl KA, Vishwasrao HD, Bailey CH, Kandel ER (2013) A single Aplysia neurotrophin mediates synaptic facilitation via differentially processed isoforms. Cell reports 3 (4):1213-1227

Kosaka N, Kodama M, Sasaki H, Yamamoto Y, Takeshita F, Takahama Y, Sakamoto H, Kato T, Terada M, Ochiya T (2006) FGF-4 regulates neural progenitor cell proliferation and neuronal differentiation. Faseb J 20 (9):1484-1485

Maretto S, Muller PS, Aricescu AR, Cho KW, Bikoff EK, Robertson EJ (2008) Ventral closure, headfold fusion and definitive endoderm migration defects in mouse embryos lacking the fibronectin leucine-rich transmembrane protein FLRT3. Developmental biology 318 (1):184-193

Miyagawa T, Saito H, Nishiyama N (1993) Branching enhancement by basic fibroblast growth factor in cut neurite of hippocampal neurons. Neuroscience letters 153 (1):29-31

Monje FJ, Birner-Gruenberger R, Darnhofer B, Divisch I, Pollak DD, Lubec G (2012) Proteomics reveals selective regulation of proteins in response to memory-related serotonin stimulation in Aplysia californica ganglia. Proteomics 12 (3):490-499

Monje FJ, Divisch I, Demit M, Lubec G, Pollak DD (2013) Flotillin-1 is an evolutionary-conserved memory-related protein up-regulated in implicit and explicit learning paradigms. Annals of medicine

Moreno H, Nadal M, Leznik E, Sugimori M, Lax I, Schlessinger J, Llinas R (1998) Nerve growth factor acutely reduces chemical transmission by means of postsynaptic TrkA-like receptors in squid giant synapse. Proceedings of the National Academy of Sciences of the United States of America 95 (25):14997-15002

Morris SA, Almeida AD, Tanaka H, Ohta K, Ohnuma S (2007) Tsukushi modulates Xnr2, FGF and BMP signaling: regulation of Xenopus germ layer formation. PloS one 2 (10):e1004. doi:10.1371/journal.pone.0001004

Nur EKA, Ahmed I, Kamal J, Babu AN, Schindler M, Meiners S (2008) Covalently attached FGF-2 to three-dimensional polyamide nanofibrillar surfaces demonstrates enhanced biological stability and activity. Mol Cell Biochem 309 (1-2):157-166. doi:10.1007/s11010-007-9654-8

Pandit SG, Govindraj P, Sasse J, Neame PJ, Hassell JR (2002) The fibroblast growth factor receptor, FGFR3, forms gradients of intact and degraded protein across the growth plate of developing bovine ribs. Biochem J 361 (Pt 2):231-241

Puthanveettil SV, Monje FJ, Miniaci MC, Choi YB, Karl KA, Khandros E, Gawinowicz MA, Sheetz MP, Kandel ER (2008) A new component in synaptic plasticity: upregulation of kinesin in the neurons of the gill-withdrawal reflex. Cell 135 (5):960-973

Shaw AE, Minamide LS, Bill CL, Funk JD, Maiti S, Bamburg JR (2004) Cross-reactivity of antibodies to actin- depolymerizing factor/cofilin family proteins and identification of the major epitope recognized by a mammalian actin-depolymerizing factor/cofilin antibody. Electrophoresis 25 (15):2611-2620

Si K, Choi YB, White-Grindley E, Majumdar A, Kandel ER (2010) Aplysia CPEB can form prion-like multimers in sensory neurons that contribute to long-term facilitation. Cell 140 (3):421-435

Skjerpen CS, Wesche J, Olsnes S (2002) Identification of ribosome-binding protein p34 as an intracellular protein that binds acidic fibroblast growth factor. The Journal of biological chemistry 277 (26):23864-23871

Szebenyi G, Dent EW, Callaway JL, Seys C, Lueth H, Kalil K (2001) Fibroblast growth factor-2 promotes axon branching of cortical neurons by influencing morphology and behavior of the primary growth cone. J Neurosci 21 (11):3932-3941. doi:21/11/3932 [pii]

van Kesteren RE, Fainzilber M, Hauser G, van Minnen J, Vreugdenhil E, Smit AB, Ibanez CF, Geraerts WP, Bulloch AG (1998) Early evolutionary origin of the neurotrophin receptor family. The EMBO journal 17 (9):2534-2542. doi:10.1093/emboj/17.9.2534

Wang W, Yang Y, Li L, Shi Y (2003) Synleurin, a novel leucine-rich repeat protein that increases the intensity of pleiotropic cytokine responses. Biochemical and biophysical research communications 305 (4):981-988. doi:S0006291X03008763 [pii]

Yamagishi S, Hampel F, Hata K, Del Toro D, Schwark M, Kvachnina E, Bastmeyer M, Yamashita T, Tarabykin V, Klein R, Egea J (2011) FLRT2 and FLRT3 act as repulsive guidance cues for Unc5-positive neurons. The EMBO journal 30 (14):2920-2933

Zhao H, Tanegashima K, Ro H, Dawid IB (2008) Lrig3 regulates neural crest formation in Xenopus by modulating Fgf and Wnt signaling pathways. Development (Cambridge, England) 135 (7):1283-1293

Zhen Y, Sorensen V, Skjerpen CS, Haugsten EM, Jin Y, Walchli S, Olsnes S, Wiedlocha A (2012) Nuclear import of exogenous FGF1 requires the ER-protein LRRC59 and the importins Kpnalpha1 and Kpnbeta1. Traffic (Copenhagen, Denmark) 13 (5):650-664
