# Supplementary figures and images for: A novel Fibroblast Growth Factor Receptor family member promotes neuronal outgrowth and synaptic plasticity in Aplysia
Source: Amino Acids. 2014 Jul 25;46(11):2477–88. doi: 10.1007/s00726-014-1803-2 (PMC4200351; doi:10.1007/s00726-014-1803-2)

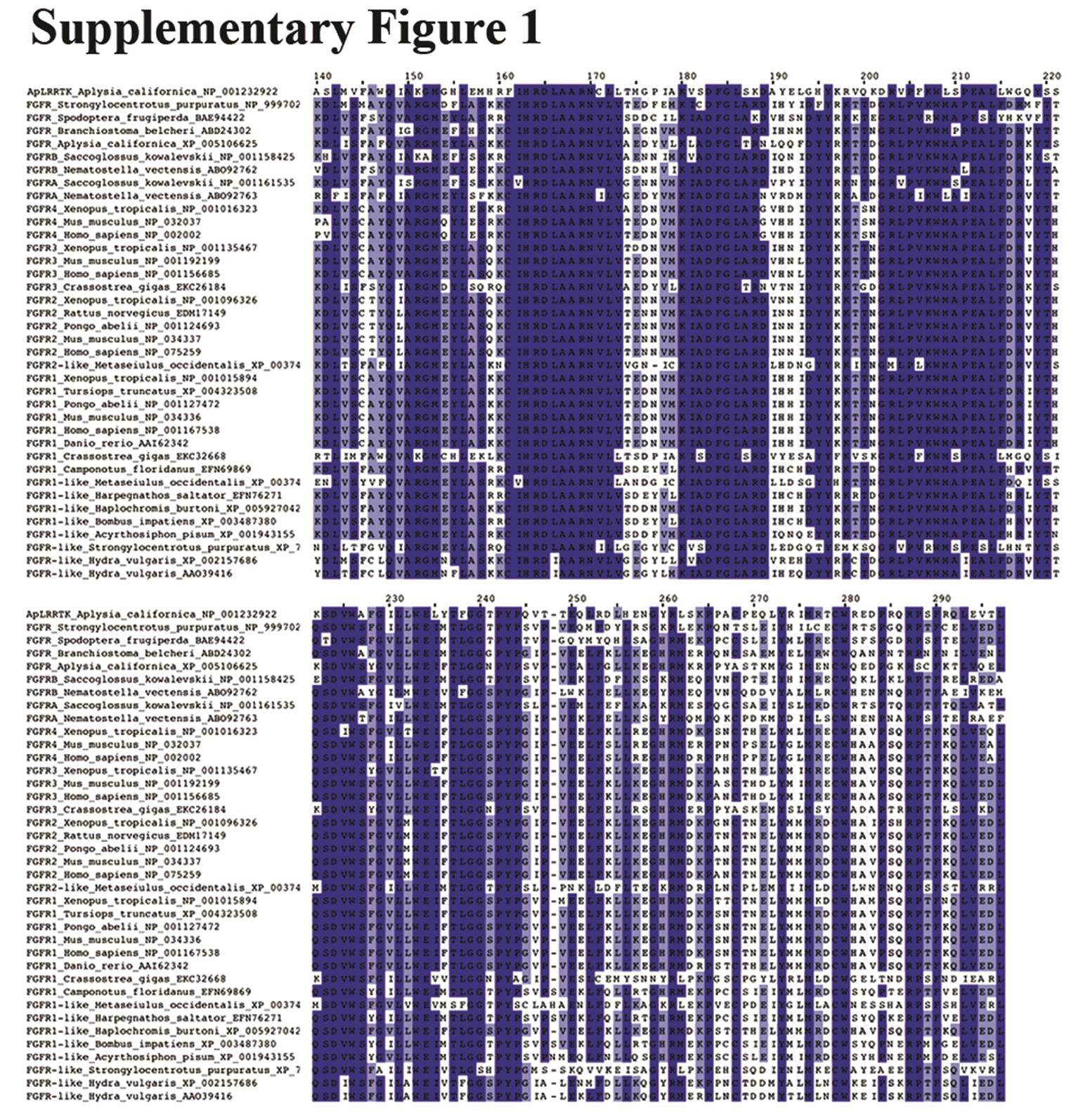

Supplement: Supplementary file 2 — Supplementary material 2 (TIFF 4687 kb) [file 726_2014_1803_MOESM2_ESM.tif]

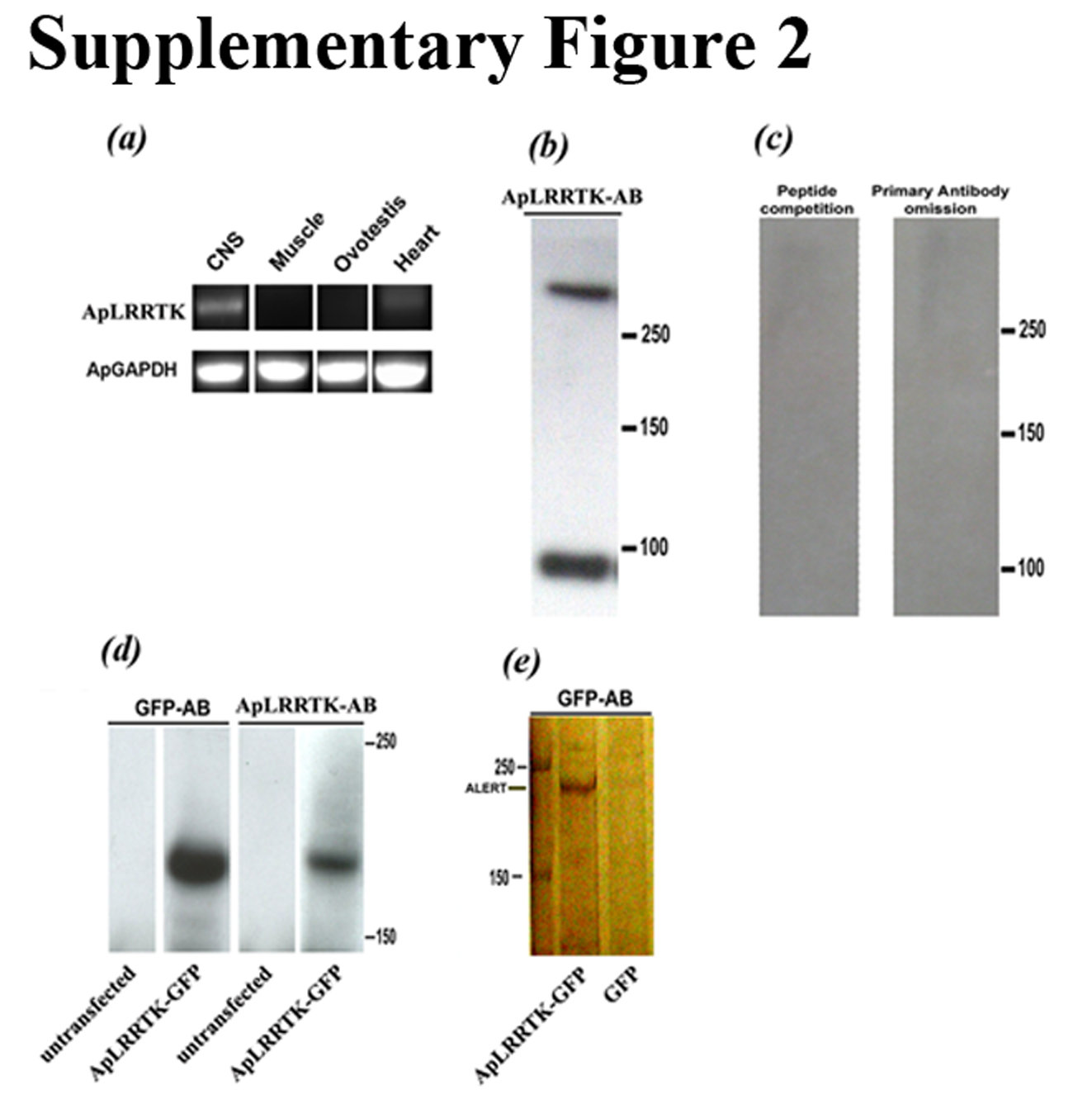

Supplement: Supplementary file 3 — Supplementary material 3 (TIFF 946 kb) [file 726_2014_1803_MOESM3_ESM.tif]

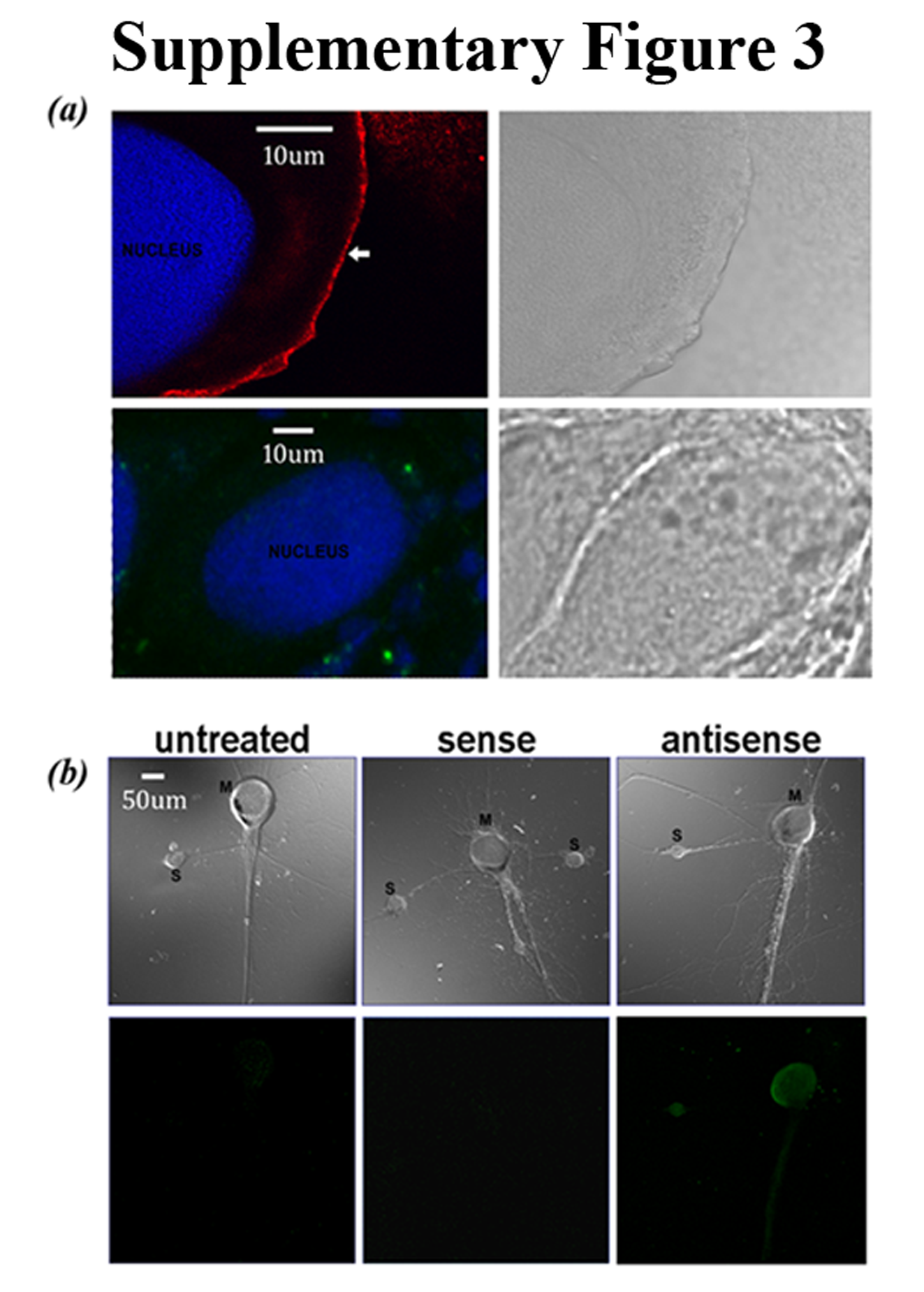

Supplement: Supplementary file 4 — Supplementary material 4 (TIFF 1726 kb) [file 726_2014_1803_MOESM4_ESM.tif]

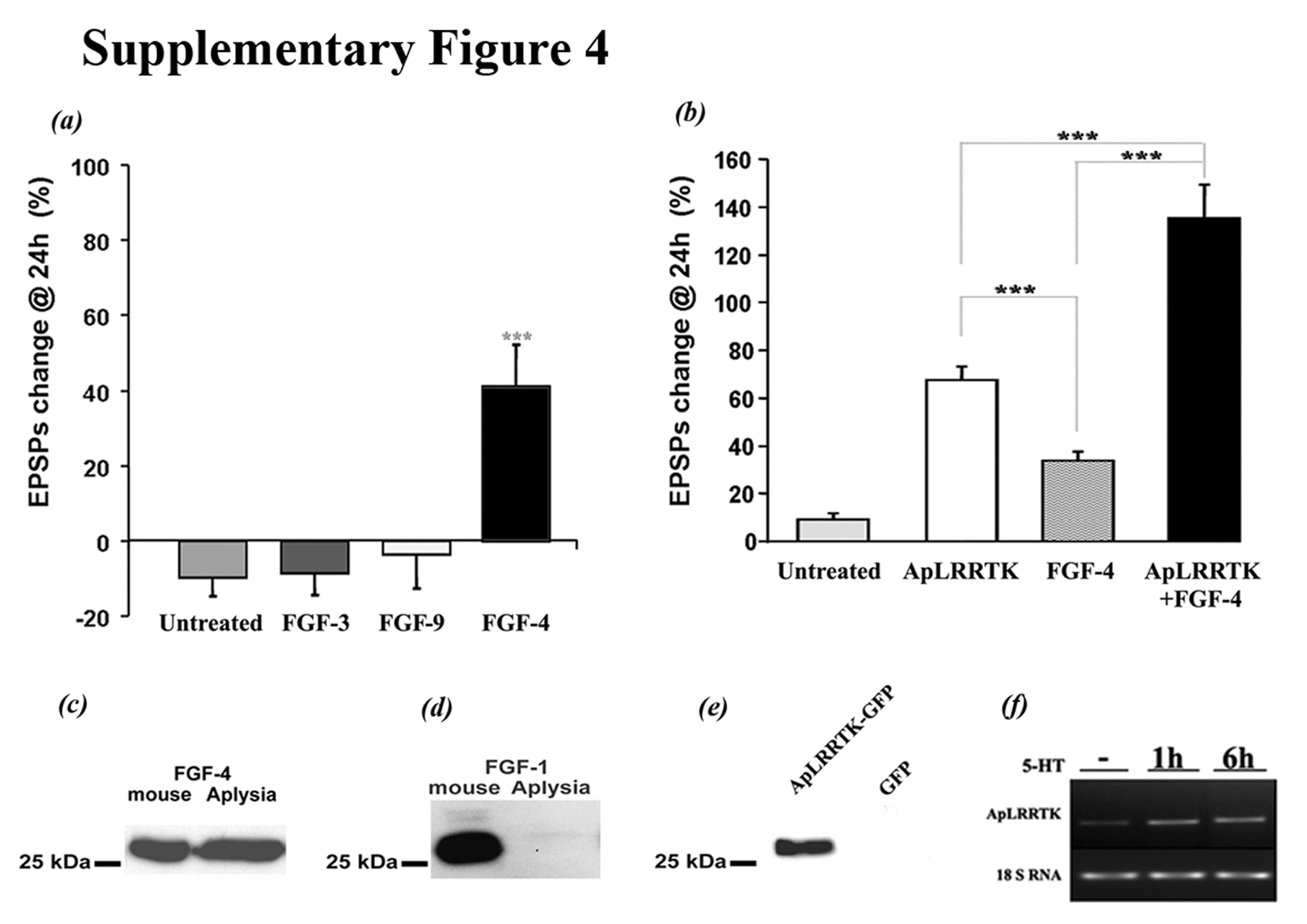

Supplement: Supplementary file 5 — Supplementary material 5 (TIFF 876 kb) [file 726_2014_1803_MOESM5_ESM.tif]

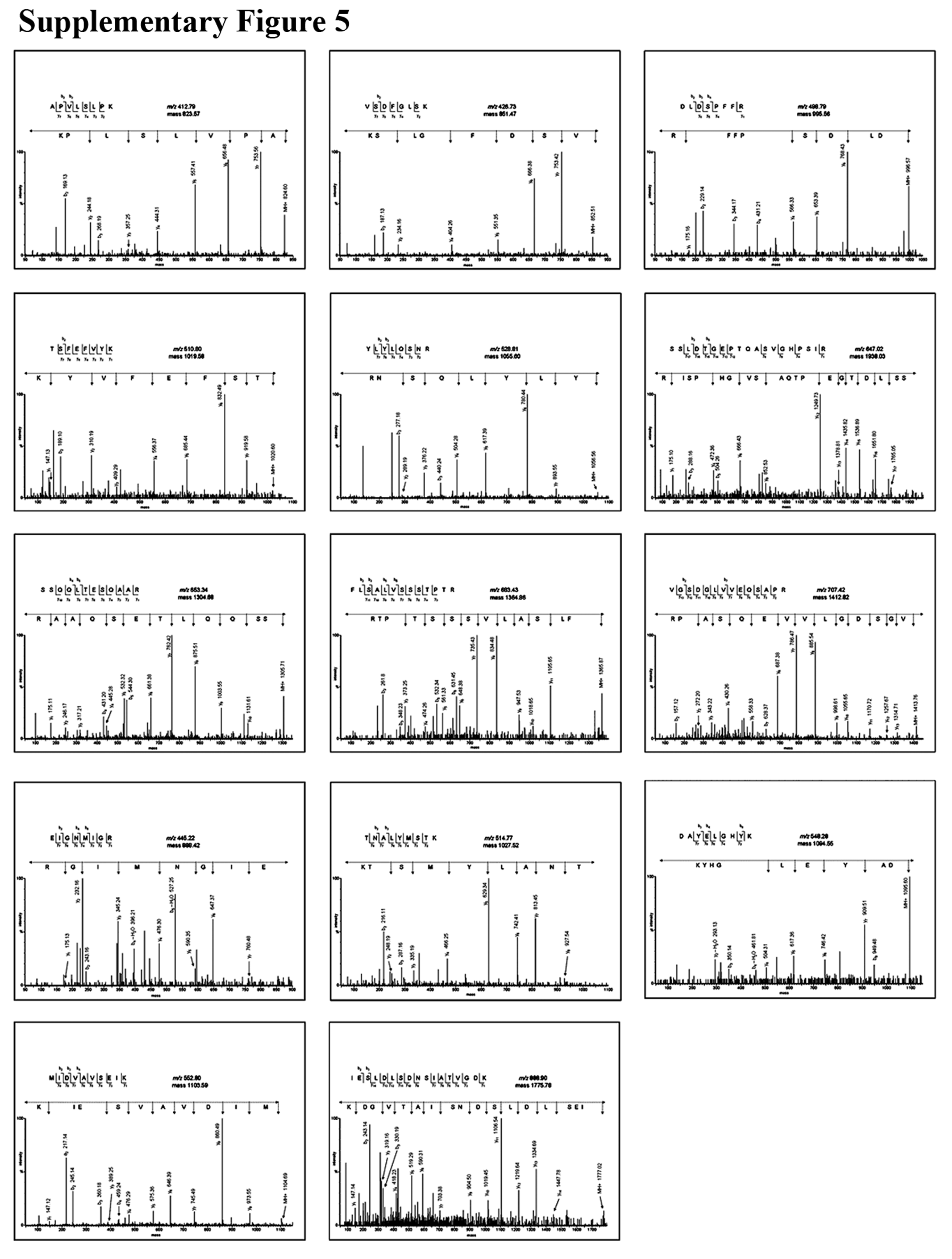

Supplement: Supplementary file 6 — Supplementary material 6 (TIFF 3434 kb) [file 726_2014_1803_MOESM6_ESM.tif]
