# Supplementary material for: A novel Fibroblast Growth Factor Receptor family member promotes neuronal outgrowth and synaptic plasticity in Aplysia
Source: Amino Acids. 2014 Jul 25;46(11):2477–88. doi: 10.1007/s00726-014-1803-2 (PMC4200351; doi:10.1007/s00726-014-1803-2)
Supplement: Supplementary file 7 — Supplementary material 7 (DOC 36 kb) [file 726_2014_1803_MOESM7_ESM.doc]

**Supplementary Table 1.**

**ApLRRTK is a member of the FGFR gene family.** from Figure 1 in the main text; lower, maximum likelihood phylogenetic tree inferred from the multiple sequence alignments for the conserved tyrosine kinase domains of representative vertebrate and invertebrate FGFRs (from Figure 1 in the main text, top to bottom correspond to FGFRs 1-38 in list the below); RETs (from Figure 1 in the main text, top to bottom correspond to RETs 39-48 in list the below); NTRK (from Figure 1 in the main text, top to bottom correspond to NTRKs 49-61 in the list below) and ROR (from Figure 1 in the main text, top to bottom correspond to ROR 62-70 in the list below).

**FGFRs family (1-38 from top to bottom)**

1. FGFR-like Strogylocentrotus purpuratus XP_794549
2. FGFR1 Crassotrea gigas EKC32668
3. **ApLRRTK Aplysia californica NP_001232922 ←**
4. FGFRB Nematostella vectensis AB092762
5. FGFR-like Hydra vulgaris AA039416
6. FGFR-like Hydra vulgaris XP_002157682
7. FGFRB Nematostella vectensis AB092763
8. FGFR3 Crassotrea gigas EKC26184
9. FGFR Aplysia californica XP_005106625
10. FGFRB Saccoglossus kowalevskii NP_001158425
11. FGFR Strogylocentrotus purpuratus NP_999702
12. FGFRA Saccoglossus kowalevskii NP_001161535
13. FGFR1-like Harpegnathos saltator EFN76271
14. FGFR1 Camponotus floridanus EFN69869
15. FGFR1-like Bombus impatiens XP_003487380
16. FGFR Spodoptera frugiperda BAE94422
17. FGFR1-like Acyrthosiphon pisum XP_001943155
18. FGFR2-like Metaseiuius occidentalis XP_003747398
19. FGFR2-like Metaseiuius occidentalis XP_003747397
20. FGFR Branchiostoma belcheri ABD24302
21. FGFR1 Pongo abelii NP_001127472
22. FGFR1 Mus musculus NP_034336
23. FGFR1 Homo sapiens NP_001167538
24. FGFR1 Tursiops truncatus XP_004323508
25. FGFR1 Xenopus tropicalis NP_001015894
26. FGFR1-like Haplochromis burtoni XP_005927042
27. FGFR1 Danio rerio AA162342
28. FGFR3 Homo sapiens NP_001156685
29. FGFR3 Mus musculus NP_001192199
30. FGFR3 Xenopus tropicalis NP_001135467
31. FGFR4 Mus musculus NP_032037
32. FGFR4 Homo sapiens NP_002002
33. FGFR4 Xenopus tropicalis NP_001016323
34. FGFR2 Xenopus tropicalis NP_001096326
35. FGFR2 Rattus norvegicus EDM17149
36. FGFR2 Mus musculus NP_034337
37. FGFR2 Pongo abelii NP_001124693
38. FGFR2 Homo sapiens NP_075259

**RETs family (39-48 from top to bottom)**

1. RET Xenopus tropicalis XP_002933941
2. RET Homo sapiens NP_066124
3. RET Mus musculus NP_033076
4. RET Aplysia californica XP_005092680
5. RET-like Metaseiulus occidentalis XP_003744478
6. RET-like Bombus impatiens XP_003487497
7. RET Camponotus floridanus EFN69712
8. RET Harpegnathos saltator EFN90166
9. RET-like Acyrthosiphon pisum XP_001947461
10. RET Drosophila melanogaster NP_477045

**NTRK family**  **(49-61 from top to bottom)**

1. NTRK2 Xenopus tropicalis NP_001072653
2. NTRK2 Mus musculus NP_001020245
3. NTRK2 Homos sapiens NP_006171
4. NTRK3 Mus musculus NP_032772
5. NTRK3 Homos sapiens NP_001012338
6. NTRK3 Xenopus tropicalis XP_002932277
7. NTRK1 Xenopus tropicalis XP_0029339035
8. NTRK1 Mus musculus NP_001028296
9. NTRK1 Homos sapiens NP_002520
10. NTRK Branchiostoma floridae AAX94284
11. NTRK1 Lymnaea stagnalis AAC26840
12. NTRK Aplysia californica NP_001232923
13. NTRK-LIKE Aplysia californica NP_001191591

**ROR family (62-70 from top to bottom)**

1. ROR2 Drosophila melanogaster NP_477255
2. ROR1 Drosophila melanogaster NP_476962
3. ROR Aplysia californica NP_001191466
4. ROR1 Mus musculus NP_038873
5. ROR1 Homo sapiens NP_005003
6. ROR1 Xenopus tropicalis XP_002931659
7. ROR2 Homo sapiens NP_004551
8. ROR2 Mus musculus NP_038874
9. ROR2 Xenopus tropicalis NP_001015834
